# Research on Assisting Clinicians to Operate rTMS Precisely Based on the Coil Magnetic Field Spatial Distribution With Magnetic Resonance Imaging Navigation

**DOI:** 10.3389/fnins.2019.00858

**Published:** 2019-08-19

**Authors:** Shijun Li, Yi Wang, ShengJie Li, Yanwei Lv, Lei Zhang, Jun Zou, Lin Ma

**Affiliations:** ^1^Department of Medical Instruments, Chinese People's Liberation Army General Hospital, Beijing, China; ^2^Department of Radiology, Chinese People's Liberation Army General Hospital, Beijing, China; ^3^Department of Stomatology, Chinese People's Liberation Army General Hospital, Beijing, China; ^4^Department of Rehabilitation, Chinese People's Liberation Army General Hospital, Beijing, China; ^5^Clinical Epidemiology and Biostatistics Research Office, Beijing Research Institute of Traumatology and Orthopaedics, Beijing, China; ^6^Department of Medical Information, Chinese People's Liberation Army General Hospital, Beijing, China; ^7^Department of Electrical Engineering, Tsinghua University, Beijing, China

**Keywords:** rTMS (repetitive transcranial magnetic stimulation), induced electric field, brain modeling, clinical teaching methodology, magnetic resonance imaging navigation

## Abstract

**Objective:** To assist clinicians to operate repetitive Transcranial Magnetic Stimulation (rTMS) precisely based on the coil magnetic field spatial distribution with Magnetic Resonance Imaging (MRI) Navigation.

**Methods:** A fast method for calculating electromagnetic fields in layered brain structures in frequency domain was proposed. By approaching Bessel function in different intervals, the integral with a highly oscillatory kernel was transformed into two parts: a definite integral and a weakened oscillatory one. The distribution of induced current density and magnetic field intensity of rTMS stimulation effect on brain was quantitatively calculated, so that clinicians could intuitively grasp the safe range of coil stimulation on the brain. Then, the crucial factor of the stimulation effect of rTMS was determined, and an accurate coil positioning of the rTMS efficiently was completed.

**Result:** The maximal attenuation of induced electric field and magnetic induction intensity was 72.20 and 86.867% at 3 cm away from the skin in the brain layered model. The clinical examination results of electric field intensity distribution, magnetic field intensity distribution, current density distribution, layered brain modeling, and coil location speed in the brain model teaching group were significantly higher than those in the traditional teaching group (*P* < 0.001).

**Conclusion:** It is suitable for clinicians to quickly complete the precise positioning of rTMS, master the adjustment of coil stimulation therapeutic parameters, and realize the precise positioning operation of rTMS with MRI navigation in intracranial.

**Clinical Trial registration:** Chinese Clinical Trial Registry (ChiCTR1800018616); Registered on 30th September 2018

## Introduction

The induced current density and the distribution of magnetic field intensity produced by repeated Transcranial Magnetic Stimulation (rTMS) acting on brain tissue is the key factor to determine its therapeutic effect of stimulation. How clinicians quickly understand these principles and grasp the accurate positioning of rTMS is crucial in making it effective. The safe range and the mechanism of therapeutic effect of coil stimulation in the accurate positioning operation of rTMS are important contents of clinical teaching, but the traditional theoretical teaching cannot vividly illustrate the advantages of this non-invasive technique. British scholar Barker et al. first used TMS to examine and evaluate the functional integrity of the central nervous conduction pathway in 1985. With the development of magnetic stimulation technology and the constant development and expansion of research, teaching, and clinical application, it has become an important nerve modulation and nerve stimulation technology. The application has gradually expanded from the initial examination and evaluation of the functional integrity of central nervous conduction pathway to the treatment of various diseases, especially the clinical intervention research on autism patients. The clinical application of rTMS in China began in the late 1980s. Technically, the accurate navigation system was not used in clinical treatment for autism patients effectively. In recent years, more and more attention have been paid to the physiological effects of rTMS on brain function. Certain effects have been achieved in the treatment of nervous system diseases such as depression (McClintock et al., 2018), central cord syndrome (Choi et al., 2019), stroke (Unluer et al., 2019), and chronic neuropathic pain (Moisset and Lefaucheur, 2019). Among them, the accuracy of stimulation is the premise of its therapeutic effect. In particular, it is very important for the selection of intracranial targets (Zhang et al., 2018) to calculate the distribution of the induced electric field positioned through the surface of the skull which is also the focus of clinical teaching.

The principle of rTMS is to induce electric field through time-varying the vmagnetic field. Specifically, a current pulse passes through the stimulation coil to generate a time-varying magnetic field, which passes through the skull and makes the adjacent nerve tissue generate a secondary current (Keuper et al., 2018). The final effect depends on the stimulation frequency, stimulation intensity, coil shape, coil direction. If the Figure-of-8 coil is utilized, the spatial resolution is about 1 cm, and the penetration depth is about 2 cm. The stimulation frequencies are different, so that the effects are also different. Low-frequency rTMS (<1 Hz) decreases the excitability of nerve cells and inhibits cortical activity, while high-frequency stimulation (5–25 Hz) increases cell excitability and enhances cortical activity (Terranova et al., 2019). Among them, stimulation accuracy is the basis for the therapeutic effect of rTMS. In order to stimulate brain cells accurately, the induced current produced by rTMS coil in various brain tissues needs to be calculated. The induced current produced by rTMS coil in the brain cannot be measured yet. In addition, the effect on brain tissue has not been taken into account in the research on the induced electric field produced by coils in the current literature.

In summary, a planar layered structure is proposed for modeling the real geometry of the brain, with the consideration of the locality property of the induced electric field. The induced current in various tissues is calculated and analyzed, so as to provide guidance for the positioning of current action points in clinical application. The main work and progress of this paper are as follows: (1) for the infinite generalized integral with double Bessel functions, a fast method of calculating frequency domain is proposed; (2) according to the time-domain induced current and the distribution of magnetic field in different brain layers, clinical learners are guided to complete the accurate positioning of rTMS quickly. (3) with the help of the simulation results and the MRI image of brain, a visualized induced electric field distribution in a different region of brain is presented, in order to aid the student to choose target points more efficiently. To the best of authors' knowledge, this is the first time a distribution of the induced electric field in the brain has been combined with an MRI image.

The study protocol approved by the Medical Ethics Committee of the Chinese PLA General Hospital (S2018-061-01) was fully disclosed to all participants and their guardians, and written informed consent was obtained from each participant's guardian according the provision of the Declaration of Helsinki. All the participants enrolled in experiments were volunteers and did not receive any reimbursement. Before the experiment, the adopted safety procedures were in line with the guidelines using rTMS (Zhang et al., 2018) and they were permitted by Chinese Clinical Trial Registry (ChiCTR1800018616).

## Methods

### Model for Calculation Electromagnetic Field With a Brain Planar Layered Structure

**Figure 1:**
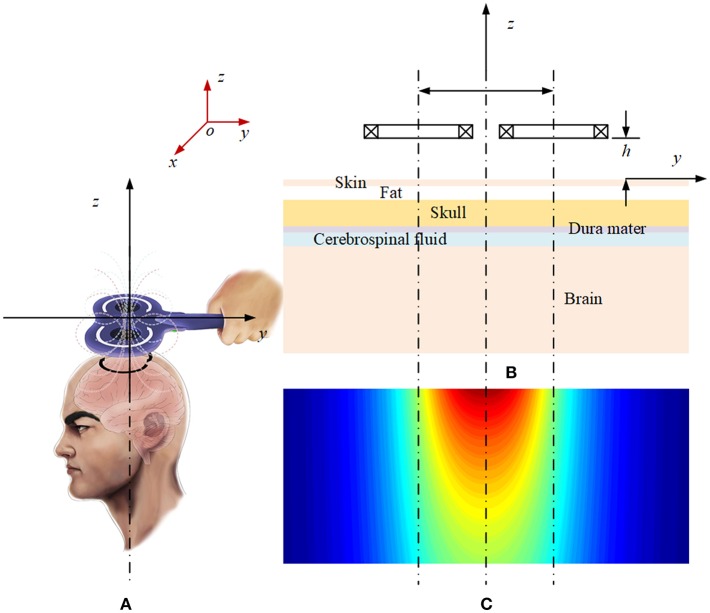
Schematic diagram of the layered plane brain model: **(A)** Magnetic field of the rTMS coil. **(B)** Profile of the layered brain model and the coils. **(C)** Magnetic field distribution on the profile plane.

**Figure 2:**
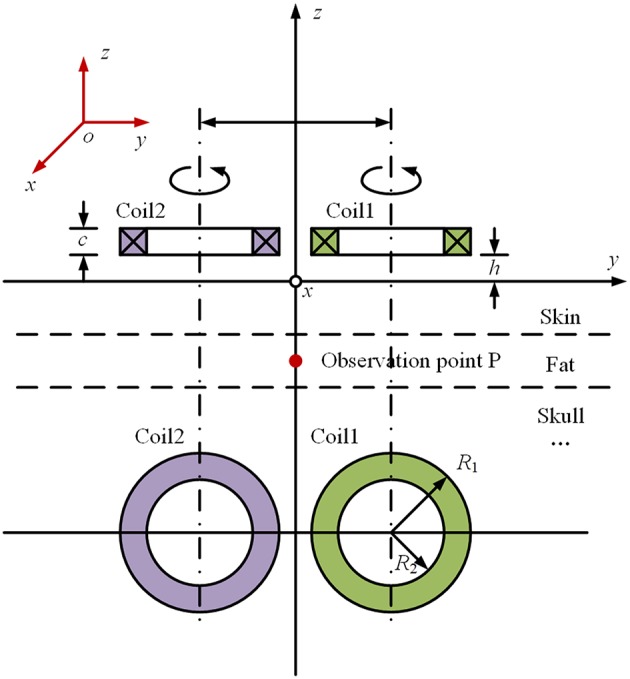
Schematic diagram of the coordinate system of “figure-of-eight coil”.

The origin of the coordinate system is set on the surface of skin, the exterior normal direction on the surface of skin is set as the positive direction of the coordinate axis, and the part below the skin is the brain tissue, which is layered according to the structure shown in Figure 1. In order to calculate the electromagnetic field generated by rTMS' figure-of-eight coil (Lu and Ueno, 2013) in the brain, a planar layered brain model is adopted (Stoecklin et al., 2016), and a global coordinate system is established, as shown in Figure 2. The distance between the coil plane and the skin is *h*, and set the total height of the coil as *c*. Figure 2 shows the profile of the two coils at the top and the top view of the two coils at the bottom. The current passing through the coils is generated by the RLC circuit (Gabriel et al., 1996). The current of the two coils is equal in magnitude and opposite in direction. The reason for using this planar layered brain model is a compromise between the mathematical simplicity and the accuracy of the field result, which will be demonstrated in the following part in the numerical example.

### Calculation of Electromagnetic Field of the Coil in Frequency Domain Using a Fast Convergent Quadature Approach

If the figure-of-8 coil is used, as shown in Figure 2, the total electric field can be calculated by superpositioning the electric field with the current flowing through each single coil. Therefore, the principal of the calculation is how to obtain the electromagnetic field in the brain tissue generated by a single coil. Take the induced electric field in the *x* direction at Point P on the Z axis in Figure 2 as an example. In frequency domain, the induced electric field in each layer of tissue can be expressed as follows (Wait, 1982):

(1) $$\begin{array}{r} E_{m}\left(\omega \right)=\text{j}\omega I\text{(}\omega \text{)}M_{m}\text{(}\omega \text{)}\;,\;m=1,2,\cdots \;,N \end{array}$$

(2) $$\begin{array}{r} M_{m}\left(\omega \right)=\frac{a}{2}\int_{0}^{\infty }F\left(\lambda \right){\text{e}}^{u_{m}z}J_{1}\left(\lambda a\right)J_{1}\left(\lambda \rho \right)\text{d}\lambda \end{array}$$

where, *E*_*m*_(ω) is the electric field generated by coil current *I*(ω) in the *m-*th layer, and *N* is the total number of layers of brain tissue. ω is the angular frequency, and *M*_*m*_(ω) is the transfer impedance between the coil current and the electric field. *a* represents the average radius of the coils. *u*_*m*_ is a variable related to the electromagnetic parameters of tissue. The definitions of ρ, *z*, and *F*(λ) are given in Appendix 1 (Wait, 1982), which includes the derivation of induced electric field of a single coil using the Hertz potential function in the cylindrical coordinate system. It is worth pointing out that the whole set of the Maxwell equation has been utilized to have a simplified equation group in (14) in Appendix 1. The Biot-Savart law cannot be used here directly to evaluate the magnetic field due to the fact that the influence of the layer media must be taken into account.

The Fourier inverse transformation is applied in Formula (1). It is easy to know according to the nature of Fourier transformation that

(3) $$\begin{array}{r} e_{m}\left(t\right)=\frac{\text{d}}{\text{d}t}i\text{(}t\text{)}⊗M_{m}\text{(}t\text{)}\;,\;m=1,2,\cdots \;,N \end{array}$$

where, *t* is the time variable, and *e*_*m*_(*t*) is the induced electric field in the *m-*th layer of tissue in time domain, *i*(*t*) is the time domain expression of the current in the coil, and *M*_*m*_(*t*) is the ⊗ impedance. is the convolution symbol.

In accordance with the constitutional relation of the *m-th* medium, the induced current density *i*_*m*_(*t*)at the observation point in the brain tissue can be calculated as follows:

(4) $$\begin{array}{r} i_{m}\left(t\right)=\sigma_{m}e_{m}\left(t\right)\;m=1,2,\cdots \;,N \end{array}$$

where σ_*m*_ is the conductivity of the *m-*th layer of brain tissue. *i*(*t*) is given by the RLC parameter of the discharge circuit. Its complete expression (Davey and Epstein, 2000) is:

(5) $$\begin{array}{r} i\text{(}t\text{)=}\frac{U_{0}}{\Omega L}\exp\left(-\alpha t\right)\sin\left(\Omega t\right) \end{array}$$

(6) $$\begin{array}{r} \alpha \text{=}\frac{R}{2L},\Omega \text{=}\sqrt{\frac{1}{LC}\left(1-\frac{R^{2}C}{4L}\right)} \end{array}$$

In which, *R, L*, and *C* are equivalent resistance, inductance and capacitance of the discharge circuit; and *U*_0_ is the initial charging voltage on the capacitor. The above parameters are known.

In order to obtain *M*_*m*_(*t*), the values *M*_*m*_(ω) at different frequencies should be calculated with Formula (2). Because the integral kernel contains the product of two Bessel functions and the brain tissue is in millimeter scale, the integral has the property of high oscillation and slow convergence, so it is difficult to calculate numerically.

Based on the asymptotic property of the Bessel function (Abramowitz and Stegun, 1965), if λ*r*>> 3/4, *J*_1_(λ*r*) can be approximated by

(7) $$\begin{array}{r} J_{1}\left(\lambda r\right)\approx \sqrt{\frac{2}{\;\pi \;\lambda r}}\cos\left(\lambda r-\frac{3}{4}\;\pi \;\right) \end{array}$$

If the truncation point *T* is determined as follows:

(8) $$\begin{array}{r} T=10\max\left(\frac{3}{4a},\frac{3}{4\rho }\right) \end{array}$$

In accordance with (7), when λ >*T*, with prosthaphaeresis formulas, a product of two Bessel functions can be converted into a sum of two sine functions, i.e.,

(9) $$\begin{array}{r} J_{1}\left(\lambda a\right)J_{1}\left(\lambda \rho \right)\approx \sqrt{\frac{\text{2}}{\;\pi \;\lambda a}}\cos\left(\lambda a-\frac{3}{4}\;\pi \;\right)\sqrt{\frac{\text{2}}{\;\pi \;\lambda \rho }}\cos\left(\lambda \rho -\frac{3}{4}\;\pi \;\right)\\ \;\text{=}\frac{\text{1}}{\;\pi \;\lambda a\rho }\left[-\sin\left(\lambda \left(a+\rho \right)\right)+\cos\left(\lambda \left(\rho -a\right)\right)\right] \end{array}$$

By substituting Formula (9) into Formula (2) and using the asymptotic property of the kernel within different intervals, three sub-integrals can be obtained,

(10) $$\begin{array}{r} M_{m}\left(\omega \right)=M_{m}^{\left(1\right)}\left(\omega \right)+M_{m}^{\left(2\right)}\left(\omega \right)+M_{m}^{\left(3\right)}\left(\omega \right) \end{array}$$

(11) $$\begin{array}{r} M_{m}^{\left(1\right)}\left(\omega \right)\text{=}\frac{a}{2}\int_{0}^{T}F\left(\lambda \right){\text{e}}^{u_{m}z}J_{1}\left(\lambda a\right)J_{1}\left(\lambda \rho \right)\text{d}\lambda \end{array}$$

(12) $$\begin{array}{r} M_{m}^{\left(\text{2}\right)}\left(\omega \right)\text{=-}\frac{\text{1}}{2\;\pi \;\rho }\int_{T}^{\infty }\frac{\text{1}}{\lambda }F\left(\lambda \right){\text{e}}^{u_{m}z}\sin\left(\lambda \left(a+\rho \right)\right)\text{d}\lambda \end{array}$$

(13) $$\begin{array}{r} M_{m}^{\left(\text{3}\right)}\left(\omega \right)\text{=}\frac{\text{1}}{2\;\pi \;\rho }\int_{T}^{\infty }\frac{\text{1}}{\lambda }F\left(\lambda \right){\text{e}}^{u_{m}z}\cos\left(\lambda \left(a\text{-}\rho \right)\right)\text{d}\lambda \end{array}$$

Formula (11) is a definite integral, whose kernel still has the product of two Bessel functions; however, the number of zeros of two Bessel functions in the interval of [0, *T*] is limited. The conventional numerical quadrature approach can be used by accumulating the integral value in each interval, which is divided the entire interval [0, *T*] according to the zeros of the Bessel function on the real axis. Formula (12) and (13) have transformed the product of double Bessel functions in Formula (2) into an integral with a single sine or cosine function, and the oscillation property of the integral kernel is effectively weakened. This is a crucial step for calculating the integral in (2), because the integral in (12) and (13) can be calculated by the steepest descent method with changing the integral path, which results in a quick convergence to obtain the integral value (Zou et al., 2014).

### Parameter Settings

For the convenience of calculation, the parameters are uniformly set as follows. According to the coordinate system shown in Figure 2, the plane of the figure-of-eight coil is parallel to the XOY plane, and its height *h* = 5 mm; and each coil has 4 turns. In order to simplify the calculation, the height difference along the Z axis of each turn of coil is not taken into account, that is *c* = 0 in Figure 2. The radius of the coil is set as 50 mm. Considering the size of cross section and winding process of the coil, there is a gap of 5 mm between the two coils. For the coil discharge circuit, the parameters are set as follows: *U*_0_ = 2.1 kV, *C* = 750 μF, *L* = 21 μH, and *R* = 0.154 Ω. The parameters for the circuit simulation are decided in accordance with the maximal current value, the decay time and rising time of the peak. For the structure of the brain tissue, one may refer to Figures 1, 2. Table 1 shows the parameters of the plane layered structure of the brain. Among them, for the conductivity data, refer to Literature. The magnetic conductivity of each tissue is set as the magnetic conductivity of vacuum.

**Table 1:** Parameters of brain tissue for the planar layered structure.

**No**.	**Tissue**	**Thickness (mm)**	**Conductivity (S/m)**
1	Skin	1.5	0.49
2	Fat	1.5	0.01
3	Skull	5	0.06
4	Dura mater	1.0	0.71
5	Cerebrospinal fluid	1.0	1.21
6	Alba	–	0.29

The brain modeling is vital to assist clinicians such as therapists or physicians to operate the rTMS precisely with Magnetic Resonance Imaging Navigation. In this paper, clinicians can quickly complete the precise positioning of rTMS with calculating electromagnetic fields in layered brain structures. The magnetic resonance navigation device is a kind of non-invasive and precise intervention for brain regions of children with autism by automatically guiding a transcranial magnetic stimulator with magnetic resonance T2 imaging. (1) Magnetic resonance imaging: three-dimensional reconstruction of the skin surface of the brain and the subject's head; display and process the corresponding three-dimensional intracranial images, image registration, fusion, reconstruction, and then registration and fusion with the stimulus coil image, and dynamic tracking of the coil position (3D image). (2) Image fusion: The fusion of intracranial 3D MR images and reconstructed extracranial 3D structures: translation, scaling, rotation, align, etc. (3) Modeling: Using ASM/AAM technology, i.e., non-rigid modeling technology, we can fit the anatomical structure of brain tissue and expand non-rigid body; after 3D image fusion of intracranial non-rigid brain tissue and extracranial rigid body bone tissue, the stimulation points of transcranial magnetic stimulator coincide with those of intracranial tissue. (4) Image registration: Using NDI three-dimensional tracking system and tracer to establish the coordinate mapping relationship between magnetic resonance image, patient's head and probe of transcranial magnetic stimulation instrument. (5) Display: Tracking prompts that the stimulation part of the coil deviates, and the space position of the coil are recalibrated based on the coil magnetic field spatial distribution, and the stimulation position of the coil is fixed. (6) Hardware: It consists of infrared emission 3D photographic head (NDI or similar products), 3D infrared reflector ball, computer, EMG amplifier, etc. (7) Software: It includes 3D image processing platform and electromyogram display system. It has the functions of processing, reconstructing, moving, overlapping and real-time monitoring of multiple 3D image objects, displaying the pattern function of motor evoked potential. Tracking shows that the stimulus position of the coil deviates from that of the coil, and recalibrates the space position of the coil and fixes the stimulus position of the coil. (8) Accessories: The magnetic resonance navigation device requires the transcranial magnetic stimulator to be equipped with a multi-coil magnetic stimulator, which can be positioned by multi-coil stereo focusing through the inverse process of electroencephalogram and fused with the magnetic resonance image data. In theory, the magnetic field of each coil can be superimposed in the focusing point to increase the depth of stimulation. The focus of stimulation decreased.

### Teaching Groups

To put forward a suitable method for clinicians to quickly complete the precise positioning of rTMS and master the adjustment of coil stimulation therapeutic parameters, which is based on the fast method for calculating electromagnetic fields in layered brain structures in frequency domain proposed, this paper assesses the difference for clinicians between one group with the traditional teaching program and the other with the brain model one.

Sixty-three graduate students of Grade 2018 of Chinese PLA General Hospital were selected, including 41 boy students and 22 girl students, with an average age of 27.9 (27.9 ± 3.92). They were randomly divided into the traditional teaching group and the brain model teaching group. The enrollment characteristics are shown in Table 2.

**Table 2:** Comparison of characteristics of enrolled students.

**Group**	**Cases**	**Age**	**Gender (M/F)**	**Degree (doctor/master)**
Traditional teaching	32	27.8 ± 3.6	20/12	11/21
Brain model	31	27.9 ± 4.4	21/10	10/21
Statistical value^*^	–	−0.090	0.190	0.032

* *Non-parametric Z-test was used for age parameters, and χ^2^ test was used for gender and student parameters*.

Two groups of students received the same 3-h teaching lecture, *Medical Imaging Technology and Clinical Application*, using the same textbook and books and different teaching forms. After the teaching and rTMS positioning operation, the clinical assessment was made.

The clinical assessment included five parts: the distribution of electric field intensity, the distribution of magnetic field intensity, the distribution of current density, layered model of brain and coil positioning speed. Each part has a full score of 20 points.

Among them, the teaching aids of the traditional teaching group include a slide projector, the paper version of teaching materials and reference books; the teaching aids of the brain model teaching group include three-dimensional real-time images of computer, an rTMS navigation device and reference books; the main lecturer of the traditional teaching group explained the slides, and asked questions about and consolidated each knowledge point for students one by one to strengthen their memory; and the main lecturer of the brain model teaching group demonstrated the induced current density and the distribution of magnetic field intensity by calculation, so that the learners could intuitively grasp the safe range of coil stimulation to the brain and understand the factors determining the therapeutic effect of rTMS stimulation to the brain.

### Statistical Method

SPSS 23.0 software was used. χ^2^ test was used to compare the disordered counting data, and the rank sum test was used to compare the ordered counting data. *P* < 0.001 indicates that the difference is statistically significant.

## Results

### Calculation of the Frequency Domain of Induced Electric Field of a Single Coil

The simulation model is built by COMSOL software, which is an axisymmetric system. The module of “magnetic field of frequency domain” in COMSOL software is used to analyze. For the sake of simplicity, the infinite boundary is set as follows: in the direction of the radius, five times of the radius of the circle is considered as the boundary, 10 times of the radius of the circle is considered as the boundary in the vertical direction, and the “magnetic insulation” condition is adopted in both directions. The brain model was set up by using data in Table 1.

The sinusoidal current with the frequencies of 0.5 kHz, 50 kHz, and 5 MHz and the effective value of 7 kA was selected as the coil current, respectively. The induced electric field of field points on the two-dimensional section line parallel to the axis in the lowest layer of the layered brain model was calculated accordingly. The relative errors are defined as follows:

(14) $$\begin{array}{r} \Delta E_{\text{P}}=100\times \frac{\left|E_{\text{P,Cal}}-E_{\text{P,SI}}\right|}{\left|E_{\text{P,Sl}}\right|} \end{array}$$

In which, *E*_p, Cal_ and *E*_P, SI_ are the induced electric field at the field points obtained by the proposed approach in this paper and the COMSOL software, respectively.

**Figure 3:**
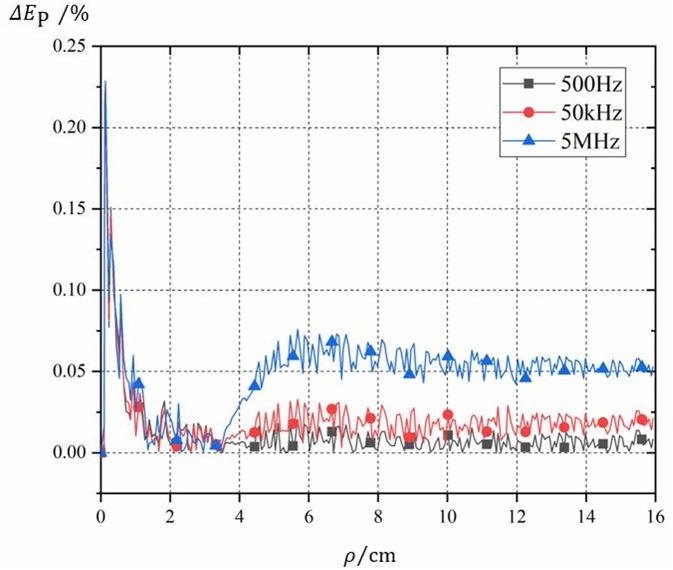
Schematic diagram of the relative error between simulation and calculation results.

Figure 3 shows the distribution of the calculation errors on the two-dimensional section line determined by Formula (14) at the selected three frequencies. As can be seen from Figure 3, the relative error between the calculation results and the simulation results is <0.25%. Figure 3 actually shows that the calculation method proposed in this paper is correct and the numerical results are accountable.

### Calculation of the Distribution of Induced Electric Field of “Figure-of-Eight Coil”

The current rTMS system can be regarded as the sequential expansion of the induced electric field generated by under-damped discharge of multiple RLC circuits in time series. Therefore, we only need to analyze the electromagnetic phenomena produced by one discharge.

**Figure 4:**
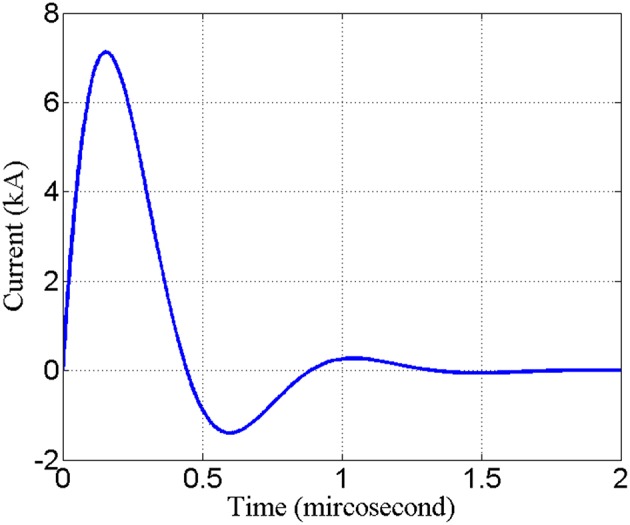
Schematic diagram of the waveform of current passing through an 8-shaped coil.

With the parameters set in this paper in the previous part, the waveform of the current passing through the “figure-of-eight coil” is shown in Figure 4. When the coil discharges once, the current duration is about 2 ms and the peak current is 7 kA.

**Figure 5:**
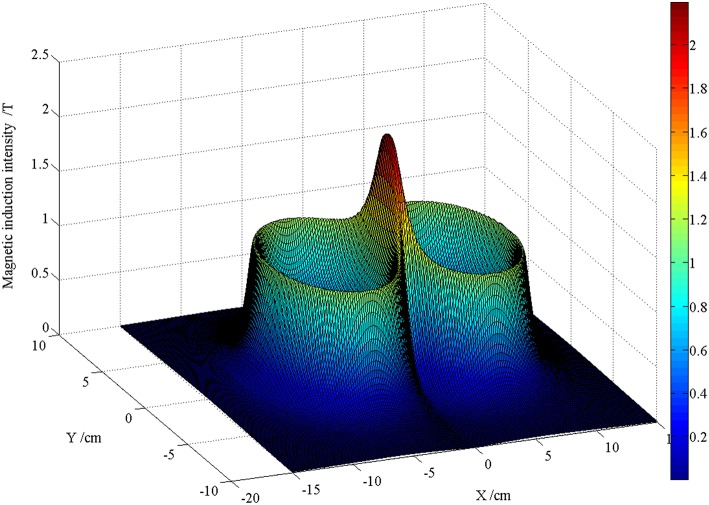
Schematic diagram of the distribution when the maximum magnetic induction intensity on the surface of skin is reached.

**Figure 6:**
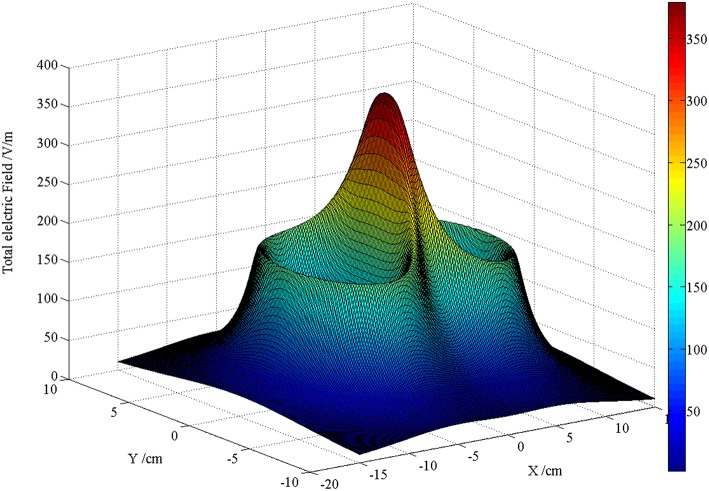
Schematic diagram of the distribution when the maximum induced electric field intensity on the surface of skin is reached.

Figures 5, 6 are the schematic diagrams of the distribution of field on the surface of skin when the magnetic induction intensity and the induced electric field intensity reach their maximum values, respectively. By referring to Figure 2, the value range on the surface of skin is X direction [−20, 20] cm and Y direction [−10, 10] cm. In the figure, the maximum magnetic induction intensity is 1.72 T and the maximum induced electric field intensity is 350 V/m. From the perspective of order of magnitude, both of them are within the range of the field quantity generated by the current rTMS system.

In Figures 5, 6, these two figures, which are pictures to reflect the spatial distribution of two kinds of field, looks alike; however, the time moment that the maximal value of two fields appears is different. Therefore, the maximal magnetic induction intensity happens when the coil current reaches its peak value, while the maximal induced electric field appears when the rising rate of current time of the coil is the maximal. From the perspective of spatial distribution, the maximal magnetic induction intensity and the induced electric field both appeared at the projection position below the center point of the “figure-of-eight coil,” which is one of the reasons that the rTMS system often uses the “figure-of-eight coil” in clinical practice to focus the effect of induced electric field.

### Calculation of the Distribution of Field in the Brain

**Figure 7:**
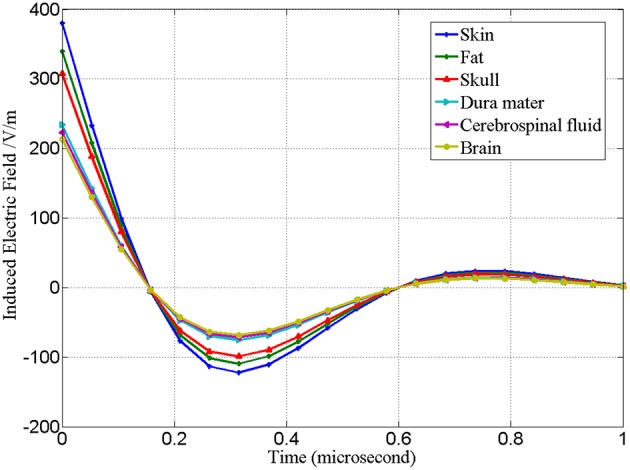
Waveform of the intensity of induced electric field of the upper surface of brain tissues at the central projection of “figure-of-eight coil” changing with time.

With coil current in Figure 4 as excitation and with reference to Figure 2, the quantity of induced electric field and induced current along Z axis at the central projection of “figure-of-eight coil” in the layered structure of the brain is analyzed. These values are the basic data for analysis on the therapeutic effect of rTMS. Figure 7 shows the waveform of the induced electric field intensity of the upper surface of brain tissues at the central projection of the “figure-of-eight coil” changing with time. Seen from the spatial distribution, the closer to the inside of the brain it is, the smaller the value of the induced electric field is. Seen from the physical characteristics of the distribution of field quantity, this result is obvious, because the farther the induced electric field from the excitation source “figure-of-eight coil” is, the smaller the numerical value will be.

**Figure 8:**
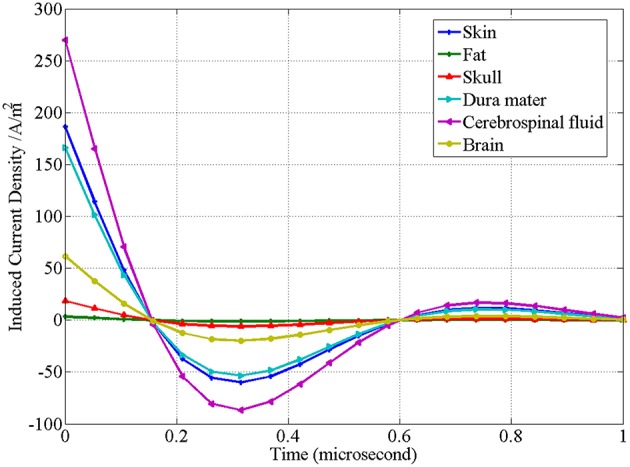
Waveform of the induced current density of the upper surface of brain tissues at the central projection of “figure-of-eight coil” changing with time.

Corresponding to Figures 7, 8 is the waveform of the distribution of induced current in various tissues changing with time. Different from the distribution of induced electric field in Figure 7, in Figure 8 the induced current density in cerebrospinal fluid is the highest, while that in fat is the lowest.

Another key issue in rTMS treatment is the electromagnetic safety of the human body. Table 3 lists the maximum induced electric field intensity of the “figure-of-eight coil” in various brain tissues. Table 3 shows the corresponding maximum magnetic induction intensity. On the basis of the induced electric field on the surface of skin, the attenuation of the induced electric field 3 cm away from the skin (corresponding to the serial number 8 in Table 3) is 72.20% on the surface of skin, and the attenuation of the magnetic induction intensity is 86.86% on the surface of skin (corresponding to the serial number 8 in Table 4).

**Table 3:** Distribution of induced electric field of rTMS coil in the layered model of brain.

**No**.	**Acting position**	**Electric field (V/m)**	**Change rate (%)**
1	Upper surface of skin	359.71	0.00
2	Upper surface of fat	326.03	−9.36
3	Upper surface of skull	297.87	−17.19
4	Upper surface of dura mater	229.75	−36.03
5	Upper surface of cerebrospinal fluid	219.33	−39.03
6	Upper surface of alba	209.66	−41.71
7	1 cm below alba	140.75	−60.87
8	2 cm below alba	100.00	−72.20
9	3 cm below alba	73.42	−79.51
10	4 cm below alba	55.16	−84.67

**Table 4:** Distribution of magnetic induction intensity of the coil in the layered model of brain.

**No**.	**Acting position**	**Magnetic induction intensity (T)**	**Change rate (%)**
1	Upper surface of skin	1.75	0.00
2	Upper surface of fat	1.46	−16.57
3	Upper surface of skull	1.23	−29.71
4	Upper surface of dura mater	0.77	−56.00
5	Upper surface of cerebrospinal fluid	0.71	−59.43
6	Upper surface of alba	0.66	−62.29
7	1 cm below alba	0.36	−79.43
8	2 cm below alba	0.23	−86.86
9	3 cm below alba	0.16	−90.86
10	4 cm below alba	0.11	−93.71

### Results of rTMS Intervention With MR Navigation

**Figure 9:**
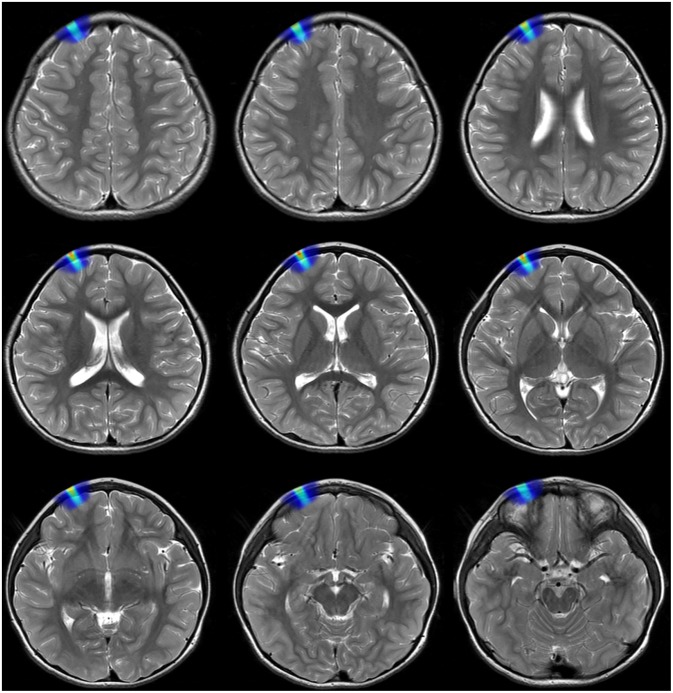
Schematic diagram of the induced electric field in different layers of brain on a preschool child with MRI in depth of 3 cm.

The dorsolateral prefrontal cortex was positioned by magnetic resonance imaging (MRI). Six preschool autistic children received treatment using the repetitive transcranial magnetic stimulator. Stereotyped movement, language disorder, social disorder, and other symptoms were initially improved. Among them, the three-dimensional teaching of the distribution of electric field intensity, the distribution of magnetic field intensity and the distribution of current density before and after intervention were adopted in the brain model teaching group (Figure 9).

### Teaching Assessment Results

The clinical assessment results of the distribution of electric field intensity, the distribution of magnetic field intensity, the distribution of current density, layered model of brain and coil positioning speed in the brain model teaching group are significantly higher than those in the traditional teaching group. The difference is statistically significant (*P* < 0.001), as shown in Table 5.

**Table 5:** Comparison of the mean score of clinical assessment between the brain model teaching group and the traditional teaching group.

**Group**	**Traditional**		**Brain modeling**		***Z***	***P***
	Med^*^	Int^*^	Med^*^	Int^*^		
Electric field	14	3	19	0	3.968	<0.001
Magnetic field intensity	14	3	20	0	3.840	<0.001
Electric current density	14	3	19	1	3.840	<0.001
Layered model of brain	15	2	18	3	3.472	<0.001
Coil positioning speed	14	2	19	2	3.968	<0.001

* *Med, Median; Int, Inter-quartile range*.

## Discussion

Figure 8 shows that the induced current density of the “figure-of-eight coil” is the highest in cerebrospinal fluid and the lowest in fat. As can be explained in Table 1 and Formula (4), the current density depends on the conductivity of the tissues and the intensity of the induced electric field acting on them. Therefore, its distribution is not simply related to the spatial position, but also closely related to the conductive properties of the tissue. In fact, the therapeutic effect of rTMS is closely related to the induced current density in tissue.

With regards to the maximum induced electric field intensity and its corresponding maximum magnetic induction intensity of the “figure-of-eight coil” in various tissues of the brain, from the upper surface of alba to 4 cm below the alba, the average change rate of the induced electric field is 10.74%, and the average change rate of the maximum magnetic induction intensity is 7.86%, which requires that the spatial accuracy of the “figure-of-eight coil” of rTMS should be at least 1 mm in clinical application.

The safety limits of induced electric field and magnetic induction intensity in the brain are beyond the scope of this study. The purpose of this paper is to give the calculation method of specific quantities of induced electric field in various tissues, so as to facilitate the rTMS system to determine reasonable irradiation intensity in practical use, and give consideration to the therapeutic effect and electromagnetic safety requirements of the human body at the same time.

This study shows that the clinical assessment scores of the brain model teaching group are higher than those of the traditional teaching group in terms of the distribution of electric field intensity, the distribution of magnetic field intensity, the distribution of current density, layered model of brain and coil positioning speed (*P* < 0.001), perhaps because space and image thinking can help with academic understanding.

In this paper, the calculation methods of electric field intensity and current density produced by the figure-of-eight coil of the rTMS system of the plane layered structure of the brain in various tissues are studied, and the corresponding calculation values are given. This method establishes a feasible channel for quantitatively determining the relationship between radiation intensity and therapeutic effect of rTMS in clinical application.

It should be pointed out that in the method in this paper, the brain is equivalent to a plane layered model. The rationality of this equivalent method needs to be further verified by physical and medical experiments. In addition, the dispersion of electromagnetic parameters of the brain has a great influence on determining the magnitude of induced currents in various tissues, which should be paid enough attention to in the follow-up study.

Of course, considering the limitation of the group of subjects, no cross-control experiment was made. The classification of doctor and master students is different between the two groups, so it is impossible to compare the advantages and disadvantages of brain model teaching for different groups. We need to improve the experimental design, and consider the effect of comprehensive factors such as cognitive level of brain imaging, cognitive level of layered model of the brain and spatial understanding ability on the results. The judgment and recording of assessment results are quite subjective, so it should be improved. Although the agreement of their new calculation method with the conventional one at a very narrow region 3 cm was shown, why the difference increases as the depth increases was not mentioned. In the conventional approach, the effect of the eddy current is omitted for the convenience of calculation, which simply leads to exaggerate the amplitude of the induced current. In this paper, all calculations first are conducted in frequency domain; i.e., and the eddy current is included naturally. In short, the eddy current is the reason to make the difference between two approaches. Admittedly, we only focus on the intervention for pre-school children of ASD with precise rTMS for they seem more urgent and meaningful than adults' if intervened by rTMS with MRI navigation effectively. Also, the number of rTMS with MRI navigation is limited in Chinese hospitals, and ASD children are the priority compared with the ASD adults. In particular, the incidence of preschool children of ASD is increasingly higher than the other ASD participants in China. So, the patients' potential demands have lead to more MRIs for pre-school children, but less for adults up to now, to some degree.

In short, through the feedback improvement of teaching, the visual image advantage of brain model teaching is obvious. In the future, we will organize long-term randomized cross-control teaching experiments, share the results of brain model, let students learn by themselves, and improve the teaching effectiveness.

## Data Availability

The raw data supporting the conclusions of this manuscript will be made available by the authors, without undue reservation, to any qualified researcher.

## Author Contributions

ShiL: conception, design of the study, acquisition, analysis, interpretation of data, and drafting the study. YW, SheL, YL, and LZ: analysis, interpretation of data, and drafting the study. JZ: technical support for the construction of the computerized task used in the study. LM: final approval of the manuscript before the submission.

### Conflict of Interest Statement

The authors declare that the research was conducted in the absence of any commercial or financial relationships that could be construed as a potential conflict of interest.
