# Supplementary material for: Research on Assisting Clinicians to Operate rTMS Precisely Based on the Coil Magnetic Field Spatial Distribution With Magnetic Resonance Imaging Navigation
Source: Front Neurosci. 2019 Aug 19;13:858. doi: 10.3389/fnins.2019.00858 (PMC6709653; doi:10.3389/fnins.2019.00858)
Supplement: Supplementary file 1 [file Data_Sheet_1.pdf]

Appendix 1:

Analytical Expression of the Induced Electric Field Produced by a Single Coil in the Planar Layered Medium

A cylindrical coordinate system is established with the surface of the layered medium model and the axis of the coil, and the schematic diagram of the layered medium model is obtained as shown in Figure 10. Among them, the coil is placed horizontally in the air. Its center coordinates are (0, 0, h), its radius is a, and the layered medium model is below the coil. It is named Layer 1, Layer 2 and so on from top to bottom. Considering the two-dimensional situation, the magnetic field generated by the rTMS coil in the layered medium has components in the ρ and z direction, while the induced electric field only has the components in the φ direction. Ignoring the displacement current, the electromagnetic field in the layered medium satisfies the system of partial differential equations.

$$\begin{cases} \dfrac{\partial E_\varphi}{\partial z} = \mathrm{j}\omega\mu H_\rho \\ \dfrac{1}{\rho}\dfrac{\partial}{\partial \rho}(\rho E_\varphi) = -\mathrm{j}\omega\mu H_z, \\ \dfrac{\partial H_\rho}{\partial z} - \dfrac{\partial H_z}{\partial \rho} = \sigma E_\varphi \end{cases} \quad (15)$$

In which, μ is the magnetic conductivity of the medium in which the field point is located, σ is the conductivity of the medium, $\omega$is the calculated angular frequency, and ρ and z are the coordinates of the field points. Considering that the object of study is the induced electric field, Formula (14) is transformed to obtain the equation only containing the electric field component.

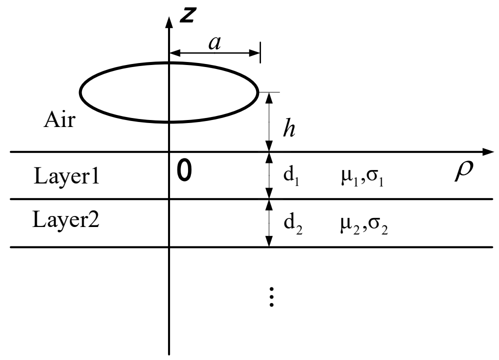

Figure 10 Schematic diagram of the coordinate system of coil in the layered medium model

$$\frac{\partial^2}{\partial \rho^2}E_\varphi + \frac{\partial^2 E_\varphi}{\partial z^2} = \mathrm{j}\omega\mu\sigma E_\varphi, \quad (16)$$

For this equation, the analytical expression of the equation is obtained by using Hertz potential.

$$E_\varphi = \mathrm{j}\omega\mu\frac{\partial F}{\partial \rho}, \quad (17)$$

In which, the expression of Hertz potential F is related to the medium layer where the field point is located.

$$F_m = \begin{cases} \dfrac{Ia}{2}\displaystyle\int_0^\infty \frac{J_1(\lambda a)}{\lambda}[\mathrm{e}^{-\lambda|z-h|} + R_0\mathrm{e}^{-\lambda(z+h)}]J_0(\lambda\rho)\mathrm{d}\lambda, m=0 \\ \dfrac{Ia}{2}\displaystyle\int_0^\infty \frac{J_1(\lambda a)}{\lambda}[A_m\mathrm{e}^{u_m z} + B_m\mathrm{e}^{-u_m z}]J_0(\lambda\rho)\mathrm{d}\lambda, m=1,2\cdots N-1 \\ \dfrac{Ia}{2}\displaystyle\int_0^\infty \frac{J_1(\lambda a)}{\lambda}A_N e^{u_m z}J_0(\lambda\rho)\mathrm{d}\lambda, m=N \end{cases} \quad (18)$$

In which, m refers to the number of medium layers, if m = 0, it is the air layer, $m = N$, it is the lowest layer of medium. $u_m = \sqrt{\lambda^2 + \mathrm{j}\omega\mu_m\sigma_m}$ (19)

On the interface of medium, the normal component of the magnetic induction intensity is continuous, and the tangential component of the magnetic field intensity is continuous, thus

$$\mu_k F_k = \mu_{k+1}F_{k+1}, \quad \frac{\partial F_k}{\partial z} = \frac{\partial F_{k+1}}{\partial z} \quad (20)$$

Suppose that the medium has a total of N layers, and the Hertz potential in each layer has 2N coefficients to be calculated. Two interfacial equations can be written for each interface of the medium, totaling 2N equations. Considering that the electric field at infinity is 0, the coefficient of exp($-u_m z$) in the Hertz potential expression of the lowest layer must be 0.Coefficients A and B are calculated from the interface of theuppermost layer

downwards in turn. Finally, all the coefficients to be determined are calculated.

Considering the derivation property of Bessel function:

$$\frac{\mathrm{d}J_0(\lambda\rho)}{\mathrm{d}\rho}=\lambda J_1(\lambda\rho), \quad (21)$$

According to Formula (20), the formula for calculating the induced electric field can be obtained from Formula (17):

$$E_0=\mathrm{j}\omega\frac{aI}{2}\int_0^{\infty}[\mathrm{e}^{-\lambda|z-h|}+R_0\mathrm{e}^{-\lambda(z+h)}]J_1(\lambda a)J_1(\lambda\rho)\mathrm{d}\lambda, \quad (22)$$

$$E_m=\mathrm{j}\omega\frac{aI}{2}\int_0^{\infty}[A_m\mathrm{e}^{u_m z}+B_m\mathrm{e}^{-u_m z}]J_1(\lambda a)J_1(\lambda\rho)\mathrm{d}\lambda, \quad (23)$$
$$m=1,2,\cdots,N-1$$

$$E_N=\mathrm{j}\omega\frac{aI}{2}\int_0^{\infty}A_N e^{u_N z}J_1(\lambda a)J_1(\lambda\rho)\mathrm{d}\lambda, \quad (24)$$

In which, R_0 is the coefficient determined according to conditions of the interface.
